# Clinical and imaging outcomes of self-locking stand-alone cages and anterior cage-with-plate in three-level anterior cervical discectomy and fusion: a retrospective comparative study

**DOI:** 10.1186/s13018-023-03726-4

**Published:** 2023-04-05

**Authors:** Liang Tang, Xiaoming Liu, Yanghu Lu, Yanbin Liu, Jiangming Yu, Jian Zhao

**Affiliations:** 1 Department of Orthopedics, Tongren Hospital, Shanghai Jiao Tong University School of Medicine, 1111 XianXia Road, Shanghai, 200336 China; 2 Department of Orthopedics, Shanghai General Hospital, Shanghai Jiao Tong University School of Medicine, 100 Haining Road, Shanghai, 200080 China; 3 Department of Orthopedics, Second Affiliated Hospital of Naval Medical University, Shanghai, 200003 China

**Keywords:** Multilevel anterior cervical discectomy and fusion, Self-locking stand-alone cage, Anterior cage-with-plate, Cervical spondylosis

## Abstract

**Background:**

Anterior cervical discectomy and fusion has been considered standard management for cervical myelopathy and radiculopathy. However, the option of using self-locking stand-alone cages or cage-with-plate in three-level anterior cervical discectomy and fusion still remains controversial. The aim of this study was to evaluate the clinical and imaging outcomes of the two procedures in multilevel anterior cervical discectomy and fusion.

**Methods:**

Sixty-seven patients who underwent three-level anterior cervical discectomy and fusion were enrolled in this study, of which 31 patients underwent surgery using self-locking stand-alone cages (group cage) and 36 patients using cage-with-plate (group plate). For the evaluation of clinical outcomes, modified Japanese Orthopedic Association scores, visual analogue scale for neck pain, neck disability index, Odom’s criteria and dysphagia status were measured. Imaging outcomes were evaluated by cervical sagittal angle, fusion segmental Cobb’s angle, fusion segmental height, range of motion, cage subsidence rate, fusion rate and adjacent segment degeneration. Statistical analyses were performed using the SPSS software (version 19.0).

**Results:**

Both groups showed improvement in modified Japanese Orthopedic Association scores, visual analogue scale for neck pain and neck disability index, after surgery, and there was no significant difference between the groups. The occurrence rate of dysphagia is significantly lower in the group cage compared with the group plate (*p* < 0.05). The postoperative cervical sagittal angle, fusion segmental Cobb’s angle, fusion segmental height and cage subsidence rate in the group plate were significantly superior to that in the group cage (*p* < 0.05). However, the rate of adjacent segment degeneration was significantly lower in the group cage compared with the group plate (*p* < 0.05). Both groups showed no significant difference in terms of fusion rate (*p* > 0.05).

**Conclusions:**

The self-locking stand-alone cages are effective, reliable and safe in anterior cervical discectomy and fusion for the treatment of cervical myelopathy and radiculopathy. Self-locking stand-alone cages showed a significantly lower rate of dysphagia and adjacent segment degeneration, while anterior cervical cage-with-plate could provide stronger postoperative stability and maintain better cervical spine alignment.

## Background

Anterior cervical discectomy and fusion (ACDF) has been considered as a standard management for cervical myelopathy and radiculopathy. ACDF with anterior plate has been clinically performed for quite a long time. Complete decompression, solid fusion and cervical lordosis recovery are the key to the success of this procedure. It was reported that ACDF with plate might result in immediate stabilization, a higher fusion rate and recovery of cervical lordosis [1, 2]. Moreover, anterior plate may also reduce the risk of graft displacement and pseudoarthrosis, particularly in multilevel cases [3, 4]. However, multilevel ACDF with a long plate means prolonged operation time, extensive exposure and traction, which might result in more complications, such as loosening of screws, trachea–esophageal injury, postoperative dysphagia, neurovascular structural injuries [5–7]. The rate of plate-related complications in multilevel ACDF even reached to 24% [5]. It was reported that the incidence of transient dysphagia after ACDF ranged from 2 to 67% within 3 months postoperatively [8–10], while the incidence of chronic dysphagia-related symptoms ranged from 3 to 21% after 3 months postoperatively [11].

Recently, self-locking stand-alone cages have been introduced in ACDF procedure to reduce the complications caused by anterior cervical plate. A study of a cohort of patients with ACDF showed that stand-alone cages led to a lower rate of dysphagia [12]. However, the major concern of the treatment with stand-alone cages is associated with high incidence of nonunion, cages subsidence, loss of cervical lordosis and pseudoarthrosis [3, 9, 13]. A meta-analysis of fusion rate in 2682 patients revealed that a higher rate of nonunion was found in cases of stand-alone cages procedures, including 7.9% in single-level ACDF, 21.1% for two-level and 35% for three-level [14]. Additionally, there was no definitive evidence whether stand-alone cages had better intermediate-term outcomes than cage-with-plate for ACDF [15]. A prospective randomized study also indicated that stand-alone Zero-p cage was not superior to conventional plate in two-level ACDF [16].

To date, the clinical efficacy and imaging outcomes between three-level ACDF using stand-alone cages and cage-with-plate still remain controversial [17]. The aim of this study was to evaluate clinical and imaging outcomes of three-level ACDF using self-locking stand-alone cages versus using cages plus anterior cervical plate (cage-with-plate).

## Patients and methods

Sixty-seven patients who received three-level ACDF from June 2017 to May 2019 were retrospectively reviewed in this study. All the included patients met the following criteria: (1) Patients with cervical radiculopathy or myelopathy. (2) Spinal cord ventral compression mainly caused by three-level cervical disk herniation on MRI. (3) Conservative treatment for at least 6 weeks was unsatisfactory. Exclusion criteria: (1) Developmental stenosis, ossification of posterior longitudinal ligament (OPLL), significant segmental instability. (2) Neck or cervical surgery history and other cervical disease, such as fracture, tumor, infection, or severe osteoporosis. Patients were divided into the group cage and the group plate, respectively, according to different surgical procedures. The group cage (ACDF with self-locking stand-alone cages) comprised 31 patients (14 male and 17 female) with a mean age of 54.6 ± 8.49 years (range from 37 to 68 years). Then, the group plate (ACDF with cage-with-plate) contained 36 patients (20male and 16 female) with a mean age of 55.7 ± 8.64 years (range from 40 to 67). The average follow-up time in the group cage and the group plate was 20.3 ± 5.6 months and 22.6 ± 3.7 months, respectively. The demographic and clinical data are collected in Table 1.

**Table 1:** Patient demographic characteristics

Variable	Group age (*n* = 31)	Group plate (*n* = 36)	*P* value
Gender(male/female)	14/17	20/16	0.762
Age (mean ± SD)	54.6 ± 8.49	55.7 ± 8.64	0.539
Course of disease	10.7 ± 3.6	11.8 ± 3.9	0.226
*Surgical level*			
C3–6	19	20	
C4–7	12	16	
Follow-up duration	20.3 ± 5.6	22.6 ± 3.7	0.17

The study conformed to the Declaration of Helsinki and was approved by the Ethics Committee of the Tongren Hospital, Shanghai Jiao Tong University School of Medicine.

### Surgical procedure

All the surgeries were operated using a standard Smith–Robinson anterior approach to the diseased levels [18]. With the guidance of C-arm, the appropriate vertebral levels were confirmed and exposed. Caspar cervical retractor was placed in the adjacent vertebral bodies. Anterior marginal osteophytes, intervertebral disk, posterior herniated disc, posterior longitudinal ligament and other compressive tissues were carefully removed to decompress the nerve roots and spinal cord completely. The upper and lower cartilage endplates were scraped completely to expose the cortical endplate. Meanwhile, the bony endplates were carefully preserved as much as possible to avoid cage subsidence. Trial cage was used to select the appropriated size.

**Fig. 1:**
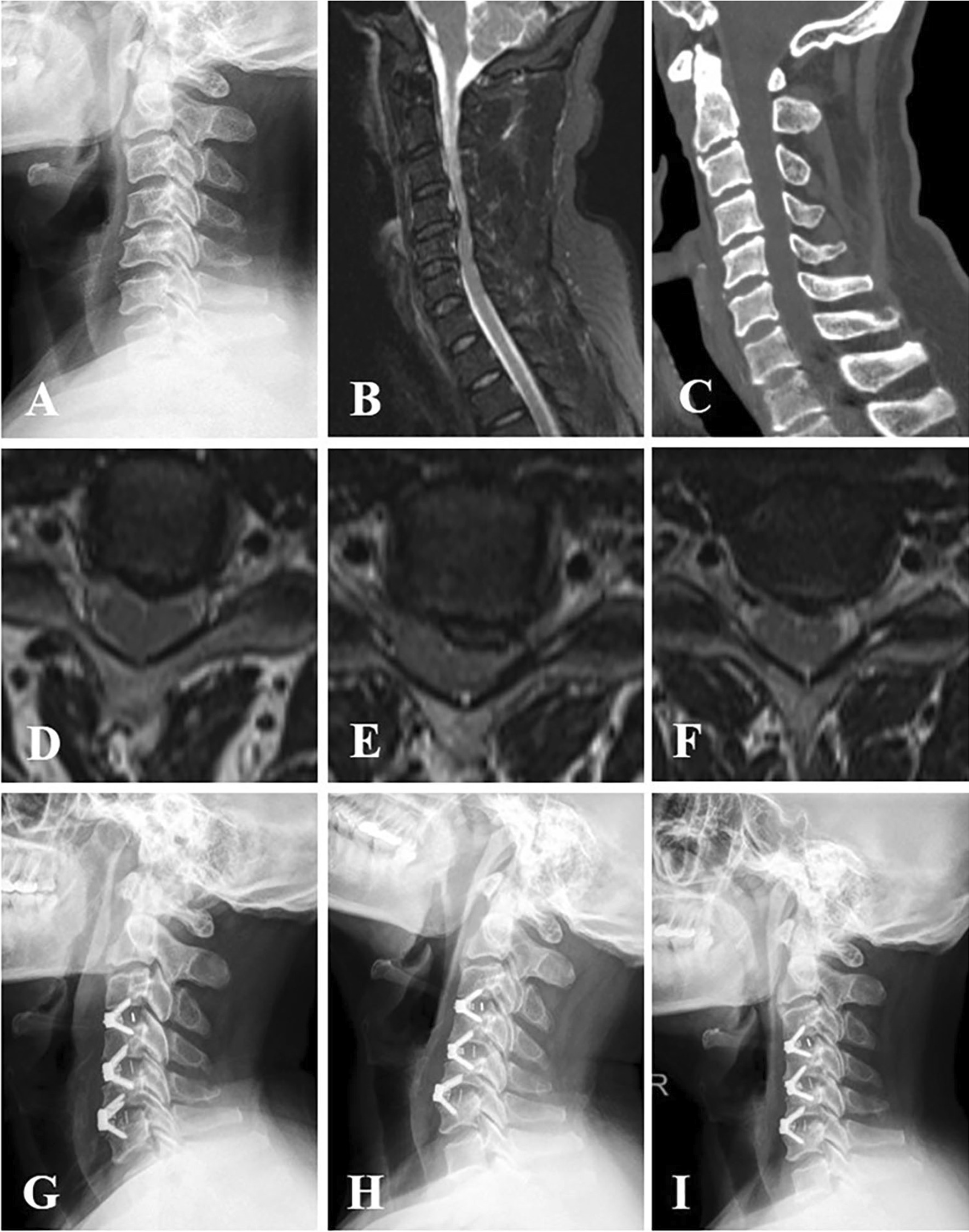
A 53-year-old man who had experienced numbness in both hands and felt stepping on cotton in lower limbs for 1.5 years was diagnosed as cervical spondylotic myelopathy. **A** Preoperative cervical lateral X-ray plain radiograph showed the alignment of cervical spine was mild kyphosis. **B**, **D**, **E**, **F** Cross-sectional and sagittal T2-weighted MRI showed degenerative disc protrusion and spinal cord compression at C3–C4, C4–C5 and C5–C6, respectively. **C** Sagittal CT reconstruction showed that no osteophyte compression was found anterior to the spinal canal. The patient who underwent three-level ACDF with stand-alone self-locking cages. **G** The X-ray examination showed the alignment of cervical spine improved significantly two days postoperatively. **H** X-ray film which was taken six months postoperatively identified that the alignment of cervical spine was well maintained, and the positions of cages were good. **I** At twelve-month follow-up, the positions of cages were still remained, whereas the lordosis of cervical spine is reduced

**Fig. 2:**
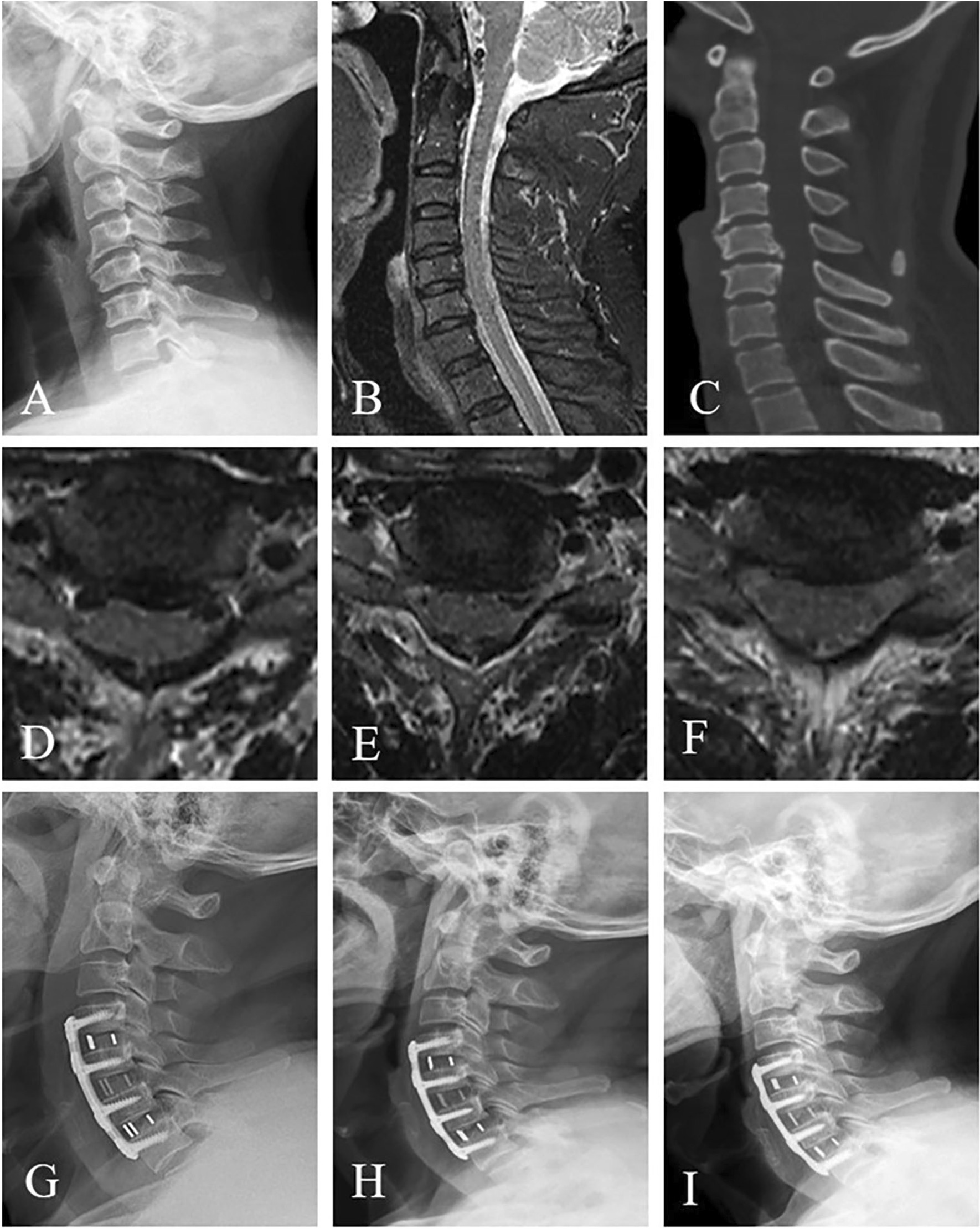
A 63-year-old man who had experienced numbness in both hands and felt stepping on cotton in lower limbs for 3 years was diagnosed as cervical spondylotic myelopathy. **A** Preoperative cervical lateral X-ray plain radiograph showed the alignment of cervical spine was straight and osteophyte emerged at the anterior edge of the vertebral body. **B, D, E, F** Cross-sectional and sagittal T2-weighted MRI showed degenerative disc protrusion and spinal cord compression at C4–C5, C5–C6, and C6–C7, respectively. **C** Sagittal CT reconstruction showed that osteophyte compression was found at C5–6 level. The patient underwent three-level ACDF with anterior cage-with-plate. **G** The X-ray examination showed that the alignment of cervical spine improved significantly two days postoperatively. **H** X-ray film which was taken five months postoperatively identified that the alignment of cervical spine was well maintained, and the positions of cages were good. **I** At fourteen-month follow-up, the lordosis of the cervical spine and the positions of cages were still remained

In the group cage, all the cages were packed with ALLOMATRIX biological material (Wright Co.) combined with autogenous bone and inserted into the disc space. Screws were inserted into the upper and lower vertebral body, respectively, through the anterior part of the cage to provide initial stabilization (Fig. 1). In the group plate, after the cages were inserted into the disc space, an anterior plate was fixed in front of the vertebral body of fusion segment (Fig. 2). All patients wore neck brace for 12 weeks postoperatively.

### Clinical evaluation

Clinical outcomes were assessed preoperatively, 3 months postoperatively and at the last follow-up. These data included visual analogue scale (VAS), modified Japanese Orthopedic Association (mJOA) score and neck disability index (NDI). The recovery rate of mJOA score was calculated according to the formula as follows: recovery rate = (postoperative score-preoperative score)/ (17-preoperative score) × 100%. Patients’ overall satisfaction was evaluated according to Odom’s criteria [19] at the end of the follow-up period. Excellent and good outcomes were considered as satisfactory.

Complications including cerebrospinal fluid (CSF) leakage, dysphagia, epidural hematoma, hoarseness, infection, implant related complications were also recorded. Dysphagia was recorded at 48 h, 2 weeks, 3, 6, 12 and 24 months postoperatively and graded as none (no episodes of swallowing problems), mild (rare episodes of dysphagia), moderate (occasional swallowing difficulty with specific food) and severe (frequent difficult swallowing with the majority of food) according to the Bazaz et al. grading system [9].

### Radiological evaluation

Radiological evaluation was analyzed in standard anteroposterior, lateral, flexion–extension radiographs preoperatively, immediately and 3 months postoperatively and the last follow-up.

Cervical sagittal angle (CSA) was determined according to the Cobb’s angle between the lower endplate of C2 and the lower endplate of C7 on lateral radiograph in neutral position. The fusion segmental Cobb’s angle (FSC) was defined by Cobb’s angle between the upper endplate of the cranial fusion segment and lower endplate of the caudal fusion segment. The fusion segmental height (FSH) was defined as the distance from the midpoint of the upper endplate to the midpoint of the lower endplate in the fusion segment. The range of motion (ROM) of the cervical spine was defined as the difference between C2–C7 Cobb’s angle on the flexion and extension radiographs. Loss of cervical lordosis, FSC, FSH and ROM were defined as the difference of the value between 3-month postoperative and the last follow-up. The fusion rate was recorded and determined by imaging findings at the final follow-up. In this study, the final follow-up time was at least 12 months. Fusion rate = amount of fusion levels/all the operation levels × 100%. Criteria of fusion on the radiographs: (1) The distance of the adjacent spinous processes between flexion and extension differs less than 2 mm; (2) No radiolucent gap was found between the graft and vertebral endplate; (3) Continuous trabecular bridging across the cage–endplate interface. Cage subsidence was described as ≥ 3 mm migration of the cage into the endplate of adjacent vertebral body on lateral plain X-rays at each time point compared to the measurement taken 2 weeks after surgery [20]. Subsidence rate = subsidence levels/all the operation levels × 100%. Adjacent segment degeneration (ASD) was assessed according to the criterion described by Kellgren and Lawrence [21] on lateral plain X-rays at the final follow-up. Radiologic findings of ASD were characterized as new osteophyte formation or enlargement, new narrowing of the intervertebral disk space or calcification of the anterior longitudinal ligament.

### Statistical analysis

Statistical analyses were performed using the SPSS version 19.0 software (SPSS Inc., Chicago, IL, USA). Baseline characteristics between the two groups were analyzed using independent t-test and Mann–Whitney U test for parametric or nonparametric variables. Radiological and clinical outcomes between the two groups were compared using repeated-measures analysis of variance. The Chi-square test was performed to evaluate the fusion rate, cage subsidence rate, the incidence of dysphagia and ASD. *P* < 0.05 was considered statistically significant.

## Results

### Clinical outcomes

All the 67 patients were followed up. The follow-up duration was 20.3 ± 5.6 months in the group cage and 22.6 ± 3.7 months in the group plate. No significant difference of follow-up duration was found between the two groups (*p* = 0.17). Nineteen patients and 12 patients underwent C3–6 and C4–7 fusion in the group cage, while 20 patients and 16 patients underwent C3–6 and C4–7 fusion in the group plate, respectively. The baseline characteristics are shown in Table 1.

The age, disease course, hospital stays, drainage were 54.6 ± 8.49 years, 10.7 ± 3.6 months, 5.97 ± 0.99 days, 58.5 ± 6.76 ml in the group cage and 55.7 ± 8.64 years, 11.8 ± 3.9 months, 6.10 ± 0.98 days, 62.1 ± 10.71 ml in group plate, respectively. There was no significant difference in age, disease course, hospital stays and drainage between the two groups (*p* > 0.05).

However, the operative time was significant shorter in the group cage (109.1 ± 24.7 min) compared with the group plate (140 ± 26.3 min) (*p* = 0.013). The blood loss was significantly less in the group cage (155.6 ± 17.3 ml) in comparison with the group plate (185.6 ± 21.8 ml) (*p* = 0.026).

Preoperative mJOA score was 7.93 ± 1.9 and 7.87 ± 2.1 in the group cage and the group plate, respectively. The mJOA score was significantly improved to 14.62 ± 1.1 at 3-month follow-up and 14.58 ± 0.8 at final follow-up in the group cage (*p* < 0.05). In the group plate, the mJOA score also increased to 14.38 ± 0.9 and 14.10 ± 1.2 at 3-month and final follow-up, respectively (*p* < 0.05). Additionally, recovery rate of mJOA score showed no significant difference between the group cage (65.12 ± 7.1) and the group plate (66.20 ± 5.9) (*p* = 0.62).

The NDI ameliorated from 17.19 ± 4.7 and 17.21 ± 5.3 preoperatively to 10.78 ± 4.16, 11.09 ± 5.23 at 3-month postoperatively, then to 9.88 ± 7.4, 9.69 ± 7.7 at final follow-up in the group cage and the group plate, respectively. Significant differences were found in both groups at 3-month and final follow-up compared with preoperative NDI (*p* < 0.05).

Preoperative VAS in the group cage and the group plate was 6.17 ± 1.8 and 6.31 ± 2.1, respectively. There was significant difference at 3-month (4.08 ± 0.99) (*p* < 0.05), final follow-up (3.58 ± 2.43) (*p* < 0.05) compared with preoperative VAS in the group cage and at 3-month (4.19 ± 1.76) (*p* < 0.05), final follow-up (3.49 ± 1.95) (*p* < 0.05) compared with preoperative VAS in the group plate.

Although the mJOA score, NDI and VAS were significantly ameliorated within group after surgery, there was no significant difference between the two groups at each time point (*p* > 0.05). According to the Odom criteria, the rate of patients who had excellent and good clinical outcomes was 93.5% and 91.7% in the group cage and the group plate, respectively, with no significant difference (*p* = 0.847).

Two patients (6.5%) in the group cage complained of moderate dysphagia 4 days after surgery. In the group plate, 5 patients had dysphagia postoperatively, of which 3 patients suffered severe dysphagia at 5th day post-op and 2 patients had moderate dysphagia at postoperative day 3, respectively. The two patients in the group cage and two patients in the group plate recovered within 2 weeks, while three patients in the group plate still remained moderate dysphagia at final follow-up. The differences of dysphagia rate between the two groups showed remarkable difference. (*p* = 0.035).

There were no occurrences of epidural hematoma, hoarseness, infection in any group. Additionally, no implant dislodgement, malposition or hardware breakage was found during the follow-up duration. CSF leakage occurred after an intraoperative dural tear due to tight adhesion and occurred in one case (1/31) of the group cage and 2 cases (2/36) of the group plate. There were no significant differences between the two groups in terms of CSF leakage (*p* = 0.566). All the clinical results are shown in Tables 2 and 3.

**Table 2:** Clinical outcomes

Variable	Group cage (*n* = 31)	Group plate (*n* = 36)	*P* value
Operation time (min)	109.1 ± 24.7	140 ± 26.3	0.013*
Blood loss (ml)	155.6 ± 17.3	185.6 ± 21.8	0.026*
Hospital day (d)	5.97 ± 0.99	6.10 ± 0.98	0.538
Drainage (ml)	58.5 ± 6.76	62.1 ± 10.71	0.071
*mJOA score*			
Preoperative	7.93 ± 1.9	7.87 ± 2.1	0.51
Immediate postoperative	13.27 ± 0.7	13.56 ± 0.6	0.761
3 months postoperative	14.62 ± 1.1^#^	14.38 ± 0.9^#^	0.736
Final follow-up	14.58 ± 0.8^#^	14.10 ± 1.2^#^	0.451
Recovery rate of mJOA score (%)	65.12 ± 7.1	66.20 ± 5.9	0.62*
*NDI*			
Preoperative	17.19 ± 4.7	17.21 ± 5.3	0.803
Immediate postoperative	14.54 ± 5.2	14.76 ± 4.7	0.695
3 months postoperative	10.78 ± 4.16^#^	11.09 ± 5.23^#^	0.657
Final follow-up	9.88 ± 7.4^#^	9.69 ± 7.7^#^	0.787
*VAS*			
Preoperative	6.17 ± 1.8	6.31 ± 2.1	0.899
Immediate postoperative	4.33 ± 1.38	4.25 ± 1.29	0.737
3 months postoperative	4.08 ± 0.99^#^	4.19 ± 1.76^#^	0.766
Final follow-up	3.58 ± 2.43^#^	3.49 ± 1.95^#^	0.753
*Odom’s criteria*			
Excellent and good rate	93.5% (29/31)	(91.7%) 33/36	0.847
*Dysphagia*			
< 3 months	6.5% (2/31)	13.9% (5/36)	0.035*
> 3 months	0	8.3% (3/36)	0.046*
Severe	0	3	
Moderate	2	2	
Mild	0	0	

^#^Comparison with preoperative

*Comparison between the two groups

**Table 3:** Clinical complications

Variable	Group cage (n = 31)	Group plate (n = 36)	*P* value
CSF leakage	3.2% (1/31)	5.6% (2/36)	0.566
Epidural hematoma	0	0	–
Hoarseness	0	0	–
Infection	0	0	–
*Implant related complications*			
Implant dislodgement	0	0	–
Implant malposition	0	0	–
Hardware breakage	0	0	–

### Imaging outcomes

In the group cage, the CSA was 10.4 ± 3.2° preoperatively, 15.9 ± 2.6° at 3-month postoperatively and 13.6 ± 5.1° at final follow-up, whereas the CSA was 10.6 ± 2.4° preoperatively, 22.1 ± 3.2° at 3-month postoperatively and 20.8 ± 7.5° at final follow-up. The CSA was significantly improved at 3-month and at final follow-up compared with that preoperative in the two groups (*p* < 0.05). However, no significant difference was found within 3 months postoperatively between the two groups (*p* = 0.223), while the CSA at final follow-up showed significant difference between the two groups (*P* = 0.005).

FSC was significantly improved from 2.6 ± 1.4°, 3.2 ± 1.7° preoperative to 10.5 ± 3.1°, 13.2 ± 3.9° at 3-month postoperative in the group cage and the group plate, respectively (*p* < 0.05), while FSC slight decreased to 8.3 ± 4.3°, 12.8 ± 3.7° at final follow-up with significant difference in comparison with preoperative (*p* < 0.05). Additionally, there was significant difference of FSC at final follow-up between the two groups (*p* = 0.015).

FSH remarkably increased from 69.5 ± 7.5 mm, 71.3 ± 8.3 mm preoperatively to 75.9 ± 7.9 mm, 79.2 ± 7.6 mm at 3-month postoperatively in the group cage and the group plate, respectively (*p* < 0.05). At final follow-up, FSH slightly decreased to 73.3 ± 8.1 mm, 79.1 ± 7.3 mm with significant difference in comparison with that preoperatively (*p* < 0.05) and also showed significant difference between the two groups (*p* = 0.021), whereas no significant difference of FSH was found between the two groups within 3 months (*p* = 0.551).

ROM of cervical spine preoperatively and at 3-month and final follow-up was 33.4 ± 7.4°, 25.1 ± 6.2° and 23.3 ± 4.8°, respectively, in the group cage, and 32.8 ± 6.5°, 24.0 ± 6.4° and 22.1 ± 5.3°, respectively, in the group plate. There was significant difference at both 3-month and final follow-up compared with that preoperative in the group cage and the group plate, respectively (*p* < 0.05), while no significant difference was found between the two groups at any time point (*p* > 0.05).

The loss of lordosis and FSC were 2.3 ± 1.9°, 2.5 ± 1.9° in the group cage and 1.5 ± 0.6°, 1.6 ± 1.3° in the group plate, respectively, with more pronounced in the group cage than that in the group plate (*p* < 0.05). The loss of FSH was also remarkably greater in the group cage (2.7 ± 1.6) than that in the group plate (0.2 ± 1.5) (*p* = 0.032), whereas the loss of ROM showed no significant difference between the two groups (*p* = 0.055).

Fusion rate was 93.5% in the group cage and 96.3% in the group plate with no significant difference between the two groups (*p* = 0.637), while cage subsidence rate and ASD rate were 17.2%, 6.5% in the group cage and 6.5%, 19.4% in the group plate, respectively, and showed significant difference between the two groups (*p* = 0.037, *p* = 0.028). The imaging outcomes are shown in Table 4.

**Table 4:** Imagingoutcomes

Variable	Group cage (*n* = 31)	Group plate (*n* = 36)	*P* value
*CSA (°)*			
Preoperative	10.4 ± 3.2	10.6 ± 2.4	0.828
Immediate postoperative	16.6 ± 6.5	22.3 ± 7.3	0.216
3 months postoperative	15.9 ± 2.6^#^	22.1 ± 3.2^#^	0.223*
Final follow-up	13.6 ± 5.1^#^	20.8 ± 7.5^#^	0.005*
Loss of lordosis	2.3 ± 1.9	1.5 ± 0.6	0.015*
*FSC (°)*			
Preoperative	2.6 ± 1.4	3.2 ± 1.7	0.724
Immediate postoperative	10.7 ± 3.3	13.5 ± 4.1	0.628
3 months postoperative	10.5 ± 3.1^#^	13.2 ± 3.9^#^	0.661*
Final follow-up	8.3 ± 4.3^#^	12.8 ± 3.7^#^	0.015*
Loss of FSC	2.5 ± 1.9	1.6 ± 1.3	0.012*
*FSH (mm)*			
Preoperative	69.5 ± 7.5	71.3 ± 8.3	0.769
Immediate postoperative	76.1 ± 8.7	79.3 ± 7.6	0.518
3 months postoperative	75.9 ± 7.9^#^	79.2 ± 7.6^#^	0.551*
Final follow-up	73.3 ± 8.1^#^	79.1 ± 7.3^#^	0.021*
Loss of FSH	2.7 ± 1.6	0.2 ± 1.5	0.032*
*ROM of cervical spine (°)*			
Preoperative	33.4 ± 7.4	32.8 ± 6.5	0.876
Immediate postoperative	25.7 ± 6.3	24.3 ± 6.2	0.164
3 months postoperative	25.1 ± 6.2^#^	24.0 ± 6.4^#^	0.185*
Final follow-up	23.3 ± 4.8^#^	22.1 ± 5.3^#^	0.077*
Loss of ROM	2.4 ± 2.1	2.9 ± 2.3	0.055*
Fusion rate	93.5% (87/93)	96.3% (104/108)	0.637*
Cage subsidence rate	17.2% (16/93)	6.5% (7/108)	0.037*
ASD rate	6.5% (2/31)	19.4% (7/36)	0.028*

^#^Comparison with preoperative

*Comparison between the two groups

## Discussion

It is still complicated and a matter of controversy on the management of multilevel cervical myelopathy and radiculopathy [22, 23]. The study showed the radiological and clinical outcomes in three-level segment ACDF between self-locking stand-alone cages and anterior cage-with-plate. The results could be summarized in the following aspects. Firstly, the cage-with-plate might do better in maintaining the cervical global and local alignment and preventing cage subsidence. Secondly, self-locking stand-alone cages might reduce the incidence of ASD compared to cage-with-plate. There was no significant difference in fusion rate between the two groups. Thirdly, clinical results showed no significant difference between the two groups in overall. Meanwhile, cage-with-plate might lead to some degree of dysphagia.

Fusion is the main concerning point in the ACDF procedure. It was reported that the fusion rate decreased with the increasing of fused segments, even additional anterior cervical plate applied [24]. Biomechanical studies demonstrated that self-locking stand-alone cages might provide similar stability as cage-with-plate in one or two-level ACDF. However, in 3- or more-level ACDF, cage-with-plate could provide more support than self-locking stand-alone cages [25]. Therefore, anterior cervical cage-with-plate could provide initial stability, avoid undesirable micro movement and has become the priority option in three-level ACDF. A literature review of 25 studies including overall 2682 patients documented that fusion rate significantly increased to 92.1% when a supplemental anterior plate has been applied for either single level or multilevel ACDF procedures [14]. Although the fusion rate was 96.3% in the group plate, which was higher than that in the group cage (93.5%), no significant difference was found between the two groups (*p* > 0.05). This study demonstrated that self-locking stand-alone cages could also gain satisfactory fusion rate in three-level ACDF. Similarly, the result was consistent with the previous reports, which showed that fusion rate of self-locking stand-alone cages ranged from 90.5 to 100% in multilevel ACDF [26, 27].

Cage subsidence is another clinical concern since cages has been applied in ACDF. If cage subsided into vertebral body, it could decrease the height of intervertebral space and the volume of intervertebral foramen, lead to sagittal malalignment and eventually result in poor clinical outcomes and adjacent segmental disease. Cage subsidence rate and its consequences differed in literatures. The reported occurrence rate of cage subsidence with self-locking stand-alone cages varied from 0 to 61% in ACDF [28, 29]. In this study, the occurrence rate of cage subsidence was 17.2% (16/93) in the group cage, which was significantly higher than that in the group plate (6.5%; 7/108). Our results were basically consistent with literature, which suggested that anterior cervical plate played an important role in preventing cage subsidence. Additionally, cage subsidence is usually accompanied by loss of FSH. In this study, FSH was significantly increased in both groups at each postoperative time point compared with that preoperatively. Meanwhile, the loss of FSH was prominent in the group cage in comparison with the group plate (*p* = 0.032). The results suggested that rigid anterior plate could shield the mechanical contact load at the cage–bone interface; consequently, it could reduce the cage subsidence and contribute to maintain FSH. However, cage subsidence might increase the mechanical load at cage–vertebra interface, thus promoting solid fusion [30]. This might explain why the stand-alone cages could obtain a higher fusion rate in three-level ACDF.

Since the callus formation might prevent the further progress of subsidence, cage subsidence usually occurred within 3 months postoperatively [31, 32]. However, Kim et al. found that subsidence might be an inevitable course and only a radiologic phenomenon with no effect on the clinical and radiologic outcomes of the use of stand-alone cages [30]. Our results also found that mJOA score, NDI, VAS in the group cage at final follow-up showed comparable outcomes with the group plate. Compared to cage subsidence, fusion had a more significant impact on clinical efficacy.

Global cervical lordosis and FSC were also considered as important factors in maintenance of clinical outcomes. Abnormal cervical sagittal alignment would increase the distribution of load on internal fixation device and the adjacent segment, therefore increasing the incidence of internal fixation failure and ASD [33, 34]. Cage reconstructed fusion segment lordosis through its own geometrical shape with higher anterior part and lower posterior part. In this study, both groups showed remarkable improvements in CSA and FSC at each postoperative time point compared with those preoperatively, whereas both of the loss of lordosis and loss of FSC were pronounced in the group cage at final follow-up than those in the group plate (*p* < 0.05). These results might show that the anterior plate contribute to alleviate the loss of global cervical lordosis and the FSC, thereby retaining cervical lordotic curvature. Since cage subsidence would also result in loss of cervical lordosis, the anterior plate helps to prevent subsidence and maintain the global cervical lordosis [35].

ASD is a common complication after ACDF, which might need to be treated eventually. The exact mechanism of ASD still remains unknown. It was generally assumed that rigid fixation of segments might lead to ASD [2, 36]. ACDF with cage-with-plate was reported to have a higher occurrence of ASD, which would be likely to accelerate the degeneration of adjacent segments [37]. In the study of Yang, the incidence of ASD was significant lower in stand-alone cages group (1.6%) than that in cage-with-plate group (18.8%) [38]. Our study showed that the rate of ASD was 6.5% in the group cage and 19.4% in the group plate, respectively, with significant difference between the two groups (*p* < 0.05). The results might be explained by the reason that the stand-alone cages located in the intervertebral disc without protruding outside, which could minimize the irritation to the adjacent cervical structures [38]. Therefore, when the anterior cervical plate was placed closer to adjacent intervertebral disk, the rate of ASD would become higher.

Rigid fixation provided by plate is likely to increase the compensatory ROM of adjacent levels. In this study, the ROM of cervical spine was comparable between the two groups at each postoperative time point. Meanwhile, the loss of ROM between the two groups was also comparable (*p* > 0.05). Since the fusion segments’ lordosis was better maintained in the group plate than the group cage, compensatory motion of adjacent segments was correspondingly increased especially during flexion. As a result, the stress on intervertebral disk of adjacent segments increased during the movement of cervical spine, which would also cause ASD [39].

Postoperative dysphagia is a well-known complication after ACDF, especially for multilevel segments procedure. The incidence of dysphagia after 3 months postoperatively was reported range from 12.5 to 35.1% [40, 41]. It was reported that 72% of the patients suffering from acute dysphagia lasting for an average of 31 days in the early postoperative period [42]. The early acute dysphagia seems to associate with intraoperative excessive traction, soft tissue edema, esophageal injury, prevertebral hematoma, long operative time and tissue adhesions [43]. In our study, the incidence of dysphagia was significantly higher in the group plate (5/36; 13.9%) compared with the group cage (2/31; 6.5%) (*p* = 0.035) at 3-month follow-up. Moreover, the patients suffered from dysphagia in the group cage recovered more easily than those in the group plate. Presumably, early dysphagia was due to the extensive surgical exposure and longer operation time, especially in three-level ACDF. Our study showed that there are longer operation time and more blood loss in the group plate (140 ± 26.3 min, 185.6 ± 21.8 ml) than those in the group cage (109.1 ± 24.7 min, 155.6 ± 17.3 ml) (*p* < 0.05). Therefore, ACDF with cage-with-plate meant longer operation time and severer soft tissue damage, which would result in soft tissue, including esophageal, injury and a higher incidence of dysphagia.

The incidence of chronical dysphagia after ACDF ranged from 3 to 21% [10]. In the current study, three patients in the group plate (8.3%; 3/36) were still suffered from dysphagia at last follow-up, while patients in the group cage showed no dysphagia anymore. It was assumed that the anterior cervical plate directly contacts with the esophagus and will irritate it. Moreover, significant correlation between thickness of anterior locking plate and postoperative dysphagia was already demonstrated [44]. Self-locking stand-alone cages were placed posterior to the esophagus with little irritation, thus effectively reducing the incidence of long-term dysphagia [45].

There are several limitations in this study. First, it is difficult to assess fusion on plain radiographs. Therefore, potential error should be taken into account. Although CT scan can determine whether fusion or not more accurately, it is unrealistic to apply CT scan during the entire follow-up. Second, cage subsidence is also associated with bone mineral density. The relationship between cage subsidence and bone mineral density was not involved in this study. Third, the prevertebral soft tissue thickness was not measured in this study, which was also associated with dysphagia. Last, prospective, large sample and randomized studies with long-term follow-up are needed to apply to determine whether the self-locking stand-alone cages have advantage over anterior cervical cage-with-plate in ACDF.

## Conclusions

In summary, the self-locking stand-alone cages showed satisfactory outcomes in three-level ACDF as anterior cervical cage-with-plate did. There was no significant difference between the two groups in term of mJOA score, NDI, VAS, fusion rate and ROM of cervical spine. However, self-locking stand-alone cages showed significantly lower rate of dysphagia and ASD compared with cage-with-plate. The anterior cervical cage-with-plate could restore cervical global alignment, FSC, FSH and prevent cage subsidence as well. Overall, the results showed that the self-locking stand-alone cages were effective, reliable and safe for ACDF, whereas for patients with severe loss of cervical lordosis and cervical instability preoperatively, cage-with-plate might be the most appropriate choice, which could help to achieve stronger stability and maintain better cervical spine alignment postoperatively.

## Data Availability

All data generated or analyzed during this study are included in this published article.

## Abbreviations

ACDF: Anterior cervical decompression and fusion
OPLL: Ossification of posterior longitudinal ligament
VAS: Visual analogue scale
JOA: Japanese Orthopedic Association
NDI: Neck instability index
CSF: Cerebrospinal fluid
CSA: Cervical sagittal angle
FSC: Fusion segmental Cobb's angle
FSH: Fusion segmental height
ROM: Range of motion
ASD: Adjacent segment degeneration
